# Chronic Lyme Disease: An Evidence-Based Definition by the ILADS Working Group †

**DOI:** 10.3390/antibiotics8040269

**Published:** 2019-12-16

**Authors:** Samuel Shor, Christine Green, Beatrice Szantyr, Steven Phillips, Kenneth Liegner, Joseph Burrascano, Robert Bransfield, Elizabeth L. Maloney

**Affiliations:** 1 Internal Medicine of Northern Virginia, George Washington University Health Care Sciences’, 1860 Town Center Drive #G220, Reston, VA 20191, USA; 2 Family Medicine, Green Oaks Medical Center, Mountain View, CA 94040, USA; drchrisg@gmail.com; 3 Internal Medicine, Pediatrics and Adolescent Medicine, P.O. Box 549, Lincoln, ME 04457, USA; bszantyr@gmail.com; 4 Internal Medicine Private Practice 944 Danbury Road, Wilton, CT 06897, USA; sephillips18@gmail.com; 5 592 Route 22-Suite 1B, Pawling, NY 12564, USA; kbliegnermd@optonline.net; 6 Northwell Hospital Systems, Northern Westchester Hospital, Mount Kisco, NY 10549, USA; 7 Nuvance Hospital Systems, Sharon Hospital, Sharon, CT 06069, USA; 8 Superior Biologics, NY 10532, USA; burraj69@gmail.com; 9 Department of Psychiatry Rutgers, RWJ Medical School, 225 Highway 35, Ste 107 Red Bank, Monmouth, NJ 07701, USA; bransfield@comcast.net; 10 Partnership for Tick-borne Diseases Education, P.O. Box 84 Wyoming, MN 55092, USA; ptde.emaloney@gmail.com

**Keywords:** Lyme disease, *Borrelia* infection, borreliosis, chronic Lyme, CLD, untreated Lyme, neuroborreliosis, late Lyme, persistent Lyme disease, post-treatment Lyme disease syndrome (PTLDS)

## Abstract

*Objective*: Chronic Lyme disease has been a poorly defined term and often dismissed as a fictitious entity. In this paper, the International Lyme and Associated Diseases Society (ILADS) provides its evidence-based definition of chronic Lyme disease. *Definition*: ILADS defines chronic Lyme disease (CLD) as a multisystem illness with a wide range of symptoms and/or signs that are either continuously or intermittently present for a minimum of six months. The illness is the result of an active and ongoing infection by any of several pathogenic members of the *Borrelia burgdorferi sensu lato* complex (*Bbsl*). The infection has variable latency periods and signs and symptoms may wax, wane and migrate. CLD has two subcategories, CLD, untreated (CLD-U) and CLD, previously treated (CLD-PT). The latter requires that CLD manifestations persist or recur following treatment and are present continuously or in a relapsing/remitting pattern for a duration of six months or more. *Methods*: Systematic review of over 250 peer reviewed papers in the international literature to characterize the clinical spectrum of CLD-U and CLD-PT. *Conclusion*: This evidence-based definition of chronic Lyme disease clarifies the term’s meaning and the literature review validates that chronic and ongoing *Bbsl* infections can result in chronic disease. Use of this CLD definition will promote a better understanding of the infection and facilitate future research of this infection.

## 1. Introduction

Lyme disease, resulting from an active infection with any of several pathogenic members of the *Borrelia burgdorferi sensu lato* complex (*Bbsl*), often affects multiple systems. It is the most common vector-borne illness in the United States [1] and Europe [2]. The Centers for Disease Control and Prevention (CDC) estimates that the annual incidence of Lyme disease in the United States exceeds 329,000 [3].

It is well-documented that many patients present with manifestations of late disease prior to receiving antibiotic therapy and investigators in the field have long known that the illness can be chronic [1,4,5,6]. While a history of a known blacklegged tick bite or erythema migrans (EM) rash allows for a timely diagnosis, few patients were aware of a tick bite prior to infection [7,8] and the incidence of EM rashes varies by geographic location and Borrelial species such that some patients never develop an EM [1,8,9]. Thus, chronic manifestations of Lyme disease may result from diagnostic delays. Chronic manifestations of Lyme disease may also result from failed antibiotic therapy as commonly prescribed regimens can be non-curative [4,10,11,12,13,14,15]. Researchers have documented that patients with acute and/or long-standing Lyme disease frequently remain ill for prolonged periods of time following treatment and that some experience disease progression despite treatment [4,15,16,17,18].

Chronic manifestations of Lyme disease are associated with significant and long-standing quality-of-life (QoL) impairments in some patients [16,17,18,19,20]. QoL scores of participants in the four National Institutes of Health (NIH)-sponsored Lyme disease retreatment trials were consistently worse than those of healthy populations [16,17,18]. In two of these trials, persistent symptoms were of such severity that they interfered with daily functioning [17]. Patients in a third trial had pain levels on par with postsurgical patients, fatigue comparable to that of multiple sclerosis patients and physical functioning similar to patients with congestive heart failure [16]. A detailed table of quality of life impairments in the NIH subjects was included in the 2014 ILADS treatment guidelines [12]. Additionally, although post-mortem determination of cause of death can be challenging, there have been reported fatalities in which *B. burgdorferi* infection was the underlying cause of death [21,22,23,24].

The economic impact of chronic manifestations of Lyme disease can be substantial. Survey responses from patients diagnosed with Lyme disease (based on CDC surveillance case criteria) who had been ill for 6 or more months, found that 39.4% and 28.3%, respectively, stopped or reduced their work hours or role and 37.3% spent at least $5000 on Lyme-related out-of-pocket expenses [25]. A study employing a medical insurance claims database also documented the financial consequences of chronic manifestations [26]. Of the 52,795 individuals diagnosed and treated for Lyme disease, total costs over a 12-month post-treatment period for patients who had one or more post-treatment Lyme disease symptoms were $3798 higher than for those who had none.

Despite the significant impact that chronic manifestations of Lyme disease can have on individuals, their families and the economy, there remains no widely accepted definition of chronic Lyme disease (CLD). A recently proposed definition divides CLD into two categories, treated and untreated [27]. The International Lyme and Associated Diseases Society (ILADS) generally agrees with that approach. Other authors proposed using the term Lyme-MSIDS (Multiple Systemic Infectious Disease Syndrome) for patients who were previously labeled as having either chronic Lyme disease or post-treatment Lyme disease syndrome (PTLDS) [28]. The purpose of this paper is to establish the International Lyme and Associated Diseases Society’s definition of chronic Lyme disease. Our immediate goal for the definition is to promote a better understanding of the infection by establishing that chronic and ongoing *Bbsl* infection can result in chronic disease. Intermediate and long-term goals are to facilitate clinical research of this infection and to improve access to care for patients with chronic Lyme disease.

## 2. Chronic Lyme Disease Definition

ILADS defines chronic Lyme disease (CLD) as a multisystem illness with a wide range of symptoms and/or signs that are either continuously or intermittently present for a minimum of six months. The illness is the result of an active and ongoing infection by any of several pathogenic members of the *Borrelia burgdorferi sensu lato* complex. The infection has variable latency periods and signs and symptoms may wax, wane and migrate. CLD has two subcategories: CLD, untreated (CLD-U) and CLD, previously treated (CLD-PT). The latter requires that CLD manifestations persist or recur following treatment and are present continuously or in a relapsing/remitting pattern for a duration of six months or more. 

The definition’s required minimum six-month duration is consistent with the definitions of other chronic infections [29,30]. While CLD can be complicated by the presence of other tick-borne pathogens [31,32], the definition does not require the presence of a co-infecting pathogen. Similarly, it is important to recognize that persistent manifestations of Lyme disease following antibiotic therapy wax and wane such that an individual’s functional performance can vary significantly over time. Although many patients with persistent manifestations of Lyme disease following treatment are functionally impaired at some point in their illness, others will not meet the criteria for functional impairment [33]. Therefore, functional status is not a component of the definition. 

ILADS’ definition of CLD, although similar to the previously offered CLD definition, differs on several key points. Both definitions have two subcategories and both require that symptoms be present for a minimum of six months. Given that acute Lyme disease, by definition, is caused by pathogenic members of the *Bbsl* complex, ILADS limits the list of potential pathogens to those bacteria while the other definition appears to include other pathogens as causative agents: “CLD may be caused by any of the known pathogenic Borrelia genospecies and associated TBD pathogens including Babesia, Anaplasma, Ehrlichia, Rickettsia, Powassan virus and possibly Bartonella” [27]. In addition, the CLD-T definition is said to describe patients who were previously treated for TBDs yet have “functionally significant fatigue, musculoskeletal pain, cardiovascular disease, and/or neuropsychiatric dysfunction that persists for six months or more.” In contrast, the ILADS definition of CLD-PT requires prior treatment specifically for Lyme disease, functional impairment is not required, and all of the known manifestations of Lyme disease can fulfill the definition. With regard to the proposed Lyme-MSIDS framework, we agree that many individuals infected with a pathogenic *Bbsl* species also may have or develop multiple systemic issues that may confound the clinical picture, but in the collective experience of this working group, many do not. Like the presence of co-existing infections, when these confounding issues are present, they are clinically important, but they are not required for the definition of chronic Lyme disease.

## 3. Microbiology

CLD may be caused by any one of several known pathogenic species in the *Bbsl* complex [34,35,36,37,38,39,40,41,42,43,44,45,46,47,48,49,50]. In the United States, Lyme disease is primarily caused by *Borrelia burgdorferi sensu stricto (Bbss).* In Europe, *Borrelia afzelii, Borrelia garinii* and *Bbss* cause the majority of cases [32]. Additional *Bbsl* species are known to cause Lyme-like illnesses but the pathogenic capabilities of other *Bbsl* species have not been fully characterized [43,51,52,53,54,55,56,57]. Please see Appendix A for a list of identified *Bbsl* species and their status as a human pathogen. Unlike the *Bbsl* pathogens, *Borrelia miyamotoi*, a member of the relapsing fever group of Borrelia, is associated with recurrent fevers and rarely produces erythema migrans lesions [58]. *Bbsl* genospecies and strains within a given species differ in terms of expressed antigens, disease presentations and response to antibiotics [42,49,59,60]. These differences introduce diagnostic uncertainties and provide additional unknowns as to optimal antibiotic regimens, thereby increasing the risk of developing CLD. 

## 4. Vector

Nymphal and adult *Ixodes* ticks are the primary vectors of Lyme disease. In the United States, transmission occurs via *Ixodes scapularis* in the Eastern and Midwestern states and *Ixodes pacificus* in the western states [51], *Ixodes ricinus* is the European vector and *Ixodes persulcatus* is the Eurasian vector [61,62,63]. *Ixodes* ticks prefer wooded or brushy areas, and exposure risk is correspondingly high in these areas [64,65]. Contact with reservoir or incidental hosts, including pets, can result in tick exposure without habitat incursion. Migratory birds are responsible for long-range dispersal and transporting ticks to previously designated non-endemic locales [66,67,68]. *Ixodes* ranges are expanding, which increases the overall risk of exposure [69].

The timing of nymph and adult activity varies by climate zone [70]. Annual case reports in the USA peak during June through August, which coincides with the peak activity of nymphal ticks in the Northeast and Midwest [51]. Adult ticks are active throughout the balance of the year. 

## 5. Pathophysiologic Basis of Chronic Lyme Disease

Chronic, active infections with *Bbsl* pathogens may result from delayed diagnosis (CLD-U) or ineffective antibiotic therapy (CLD-PT), or both [71,72,73,74,75,76]. Pathogenic *Bbsl* have the ability to invade a wide variety of cells and tissues, including: fibroblasts, glial and neuronal cells, endothelial cells, lymphocytes, synovium, skin, ligaments, cardiac tissue, lymph nodes and tonsillar lymphoid tissue [77,78,79,80,81,82,83,84,85,86,87,88,89]. Pathologic examination of infected tissues correlated clinical manifestations of CLD with the invasion of these tissues [90,91,92]. 

Literature reports and studies dating back to 1979 have documented chronic and late manifestations of active infection with *B. burgdorferi* including carditis, meningitis, cranial nerve palsy, radiculopathy, arthritis, reversible peripheral neuropathies, reversible chronic encephalopathy, polyneuropathy, leukoencephalitis, cognitive and psychiatric symptoms as well as fatigue, headache, hearing loss, tinnitus and fibromyalgia [4,92,93,94,95,96,97]. Importantly, some studies used objective assessments of pathology to confirm subjective data that lacked corresponding physical exam findings [13,92].

The etiology of persistent clinical manifestations in patients previously treated for Lyme disease continues to be debated as the pathophysiologic evidence base continues to expand and evolve. Several mechanisms, including tissue injury [98], Lyme-induced secondary conditions [99,100,101,102], unrecognized or undertreated co-infections [12,98], immune dysfunction of several types [103,104,105,106,107,108], and persistent *Bbsl* infection have been proposed [12,109,110]. Types of potential post-treatment immune dysfunction include failure to clear antigenic debris [103,104], the formation of autoantibodies [105,106] and persistent elevation of immune mediators [107,108]. It is possible that more than one mechanism may be operative in a given individual.

To this working group, the volume of animal and human evidence documenting persistent *Bbsl* infection following antibiotic therapy, a requisite component of our CLD-PT definition, is substantial, and thus, quite persuasive [7,22,23,71,72,73,74,76,83,111,112,113,114,115,116,117,118,119,120,121,122,123,124,125,126,127,128,129,130,131,132,133,134,135,136,137,138,139,140]. Persistent infection has been demonstrated in patients with Lyme disease by PCR and culture [22,23,71,72,73,74,76,83,113,118,119,121,125,126,136,137,138,139,140]. A xenodiagnostic study in humans, sponsored by the NIH, documented the acquisition of B. burgdorferi DNA by uninfected ticks which fed on a persistently symptomatic patient who had been treated for Lyme disease more than 1 year earlier [129]. Given the expectation that the immune system would typically clear bacterial debris quickly, this finding is significant and strongly supports that the infection was ongoing. Animal studies have corroborated the human findings, documenting bacterial persistence by culture, PCR, histopathologic testing of post-treatment necropsy specimens and by xenodiagnoses [130,131,132].

Potential survival mechanisms of *Bbsl* persistence include: immune evasion, immune modulation, and the presence of subpopulations of persister cells. Physical seclusion—within cells [84,85,141], collagen-rich tissues [142], and immunologically protected sites (CNS, joints, and eyes) [143,144,145], is one method of immune evasion. Biofilm generation is another recognized form of physical seclusion. Published reports document that *Borrelia burgdorferi* can produce biofilm in vitro [146] and examination of infected human tissues demonstrated *B. afzelii* [147] and *B*. *burgdorferi* [148] embedded in biofilm.

Immune evasion via alterations in its physical structure may also contribute to *Bbsl* survival. Such alterations include phasic and antigenic variations [149,150,151,152,153], producing changes in the expression of outer surface proteins (Osp), and morphologic changes leading to cell-wall deficient forms, round bodies, spherocytes and “cyst” forms [154,155,156,157,158,159].

*Bbsl* pathogens can modulate the effectiveness of the host immune response via altered complement [160,161,162], neutrophil and dendritic cell functioning [163,164], alterations in the adaptive immune response [165,166,167] as well as changes in cytokine and chemokine levels [105,168,169].

In addition, several researchers have published on the existence of persister populations of *Bb* [170,171,172,173,174]. A recently developed mouse model of Lyme arthritis resulting from infection with persister microcolony forms found that this bacterial form caused more severe arthritis than log growth spirochetal forms. Microcolony infections could not be eradicated by commonly used antibiotics for Lyme [174].

Researchers have noted that manifestations often followed an intermittent, recurrent course, that disease latency varied by system, and that symptom migration within and between systems did not follow a predictable temporal pattern [4,93,94,95,96,97,136,137,175,176,177]. These observations are consistent with detailed studies of the pathogenesis of *Borrelia* infections in mammals. In mammalian models, *B. burgdorferi* rapidly developed genetic and antigenic variations beginning within days of initial infection [149,150,178]. This antigenic variation was random, induced by host factors and increased over time. Investigators concluded that the process could potentially result in “millions” [149] of variations and contribute to *B. burgdorferi*’s immune evasion capabilities and tissue tropism. Thus, this phenomenon may underlie the changing and migratory presentation of CLD.

While some have claimed a lack of therapeutic efficacy in the NIH-sponsored antibiotic retreatment trials and use this to challenge the existence of persistent *Bbsl* infections [16,17,18,98,179], ILADS and several other groups reviewing the NIH-sponsored trials of antibiotic retreatment have noted problems with trial design and execution [12,110,180,181]. Had the NIH trials been without these design flaws, valid conclusions regarding the effectiveness of the specific therapeutic regimen used in each trial could have been drawn but universal conclusions regarding the effectiveness of all antibiotic regimens are beyond the scope of those trials. Therefore, conclusions regarding infection status that are based on a lack of a therapeutic response are faulty as an absent response is not proof that the subjects were not infected. Determining infection status in these circumstances is a distinctly different task, one that requires the application of a test of bacteriological cure, which is lacking in Lyme disease. Despite this, the Krupp trial which was well-designed on its fatigue endpoint, demonstrated a sustained moderate to large treatment effect in patients with severe fatigue [18], a finding that was corroborated in a post-hoc analysis of the severe fatigue patient subset of the Fallon cohort [16].

## 6. Clinical Manifestations of Chronic Lyme Disease

### Methods

**Figure 1:**
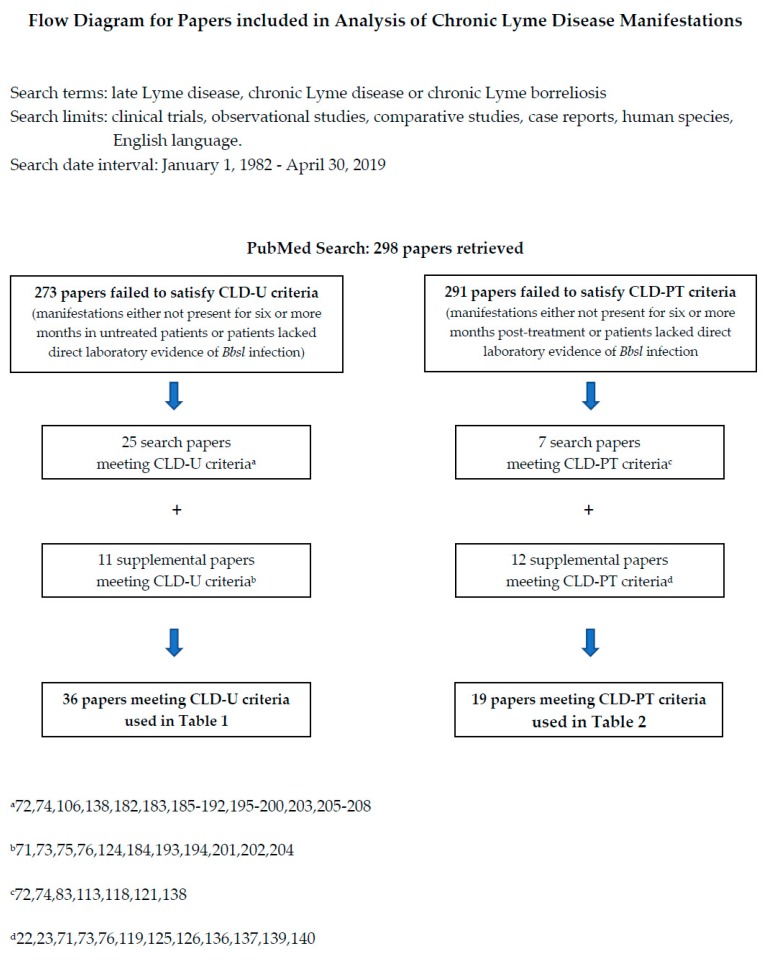
Flow Diagram of Literature Search.

To establish literature support for the ILADS definitions of CLD-U and CLD-PT and to characterize the clinical spectrum of these entities, the working group performed an electronic search of the Medline database via PubMed on 30 April 2019, using these terms—late Lyme disease, chronic Lyme disease and chronic Lyme borreliosis and these filters—clinical trials, observational studies, comparative studies, case reports, human species, English language. Two hundred and ninety-eight papers were retrieved. The search was supplemented by additional publications referenced in the retrieved documents as well as papers known to the working group (See Figure 1).

With regard to CLD-U, retrieved papers were reviewed in order to identify manifestations and/or conditions present for 6 or more months in untreated patients who had direct laboratory evidence of *Bbsl* infection (positive culture, positive PCR (polymerase chain reaction), positive antigen detection, and/or positive microscopy with *Bb*-specific immunohistochemistry). Twenty-five papers met those parameters and these were supplemented with an additional eleven papers meeting those same parameters [71,72,73,74,75,76,106,124,138,182,183,184,185,186,187,188,189,190,191,192,193,194,195,196,197,198,199,200,201,202,203,204,205,206,207,208]. With regard to CLD-PT, the retrieved papers were reviewed in order to identify manifestations that were present for six months or more post-treatment in patients who had direct laboratory evidence of *Bbsl* infection (positive culture, positive PCR, positive antigen detection, and/or positive microscopy with *Bb*-specific immunohistochemistry). Seven papers met those parameters and these were supplemented with an additional twelve papers meeting those same parameters [23,71,72,73,74,76,83,113,118,119,121,125,126,136,137,138,139,140].

The papers cited in Table 1; Table 2 do not lend themselves to a formal statistical analysis of the frequency of the various symptoms and signs. However, it is interesting to note that there is a strong overlap between the most commonly identified CLD-U and CLD-PT symptoms. The six CLD-U symptoms with the greatest number of supporting papers were arthralgia, fatigue, sensory changes (hypoesthesia/paresthesias), joint swelling, headache, and skin discoloration. The six CLD-PT symptoms with the greatest number of supporting papers were arthralgia, fatigue, headache, sensory changes (hypoesthesia/paresthesias/hypoalgesia) impaired memory, myalgia.

These findings closely mirror those of a recent community-based study of treated Lyme disease patients who were followed longitudinally [209]. The most commonly reported symptoms in the persistently symptomatic group included the common CLD-U and CLD-PT symptoms. A validated screening questionnaire for Lyme disease also substantiates the clinical relevance of the most common CLD-U and CLD-PT manifestations [177]. Furthermore, a study comparing reported symptoms in post-treatment Lyme disease syndrome (PTLDS) patients versus controls found that the rates of fatigue, pain, sleep disturbance, and depression were significantly higher and more severe in the PTLDS cohort [210].

## 7. Comparison to the Definition of Post-Treatment Lyme Disease Syndrome

Post-treatment Lyme disease syndrome (PTLDS) and post-Lyme disease syndrome (PLDS) have been used to describe patients who remain ill following antibiotic treatment for Lyme disease [33,98]. These two terms are frequently, though imprecisely, used interchangeably. Although originally proposed as an operational definition [33], PTLDS is primarily a research definition. A recently released draft of the Infectious Diseases Society of America (IDSA)/American Academy of Neurology (AAN)/American College of Rheumatology (ACR) guidelines for Lyme disease did not use PTLDS in the document, instead it discussed “prolonged symptoms following treatment of Lyme disease” [211].

The PTLDS definition is clinically more narrow than the CLD-PT definition described in this paper [33]. Although both the PTLDS and the CLD-PT definitions address ongoing post-treatment symptoms which last at least 6 months, the PTLDS definition utilizes a limited number of symptoms and more stringent exclusionary criteria. Additionally, PTLDS requires that patients have impairments in their daily functioning. Thus, while a subset of CLD-PT patients would satisfy the PTLDS definition, many would not.

It is also important to note that the PTLDS designation does not speak to the underlying mechanism(s) for ongoing symptoms while the CLD-PT definition specifically requires an ongoing *Bbsl* infection.

## 8. Limitations

The scientific understanding of chronic Lyme disease is rapidly evolving. While the pathogenic members of the *B. burgdorferi sensu lato* complex are the undisputed cause of Lyme disease, whether there is a role for other pathogens in chronic Lyme disease is unclear. This uncertainty potentially limits the inclusivity of the ILADS CLD definition as the definition does not address non-*Bbsl* pathogens. The CLD-PT subset of the definition requires an ongoing *Bbsl* infection despite antibiotic treatment for Lyme disease; it does not address residual symptoms due to non-*Bbsl* causes such as tissue injury or immune dysregulation. The lack of terminology for these entities is another limitation of the CLD definition.

The narrow focus on ongoing *Bbsl* infection makes the ILADS CLD definition suitable for research purposes (and researchers might use Table 1 and Table 2 to identify subjects who may be appropriate for their studies); this definition is not intended to serve as diagnostic criteria. No attempt was made to designate major or minor symptom-based criteria or other diagnostic schemes, which limits the definition’s clinical utility. Chronic Lyme disease, as documented in Table 1 and Table 2, has a plethora of clinical presentations and distinguishing this entity from other similarly presenting conditions, both infectious and noninfectious, can be challenging for clinicians. The situation is exacerbated by the paucity of clinically available direct diagnostic tests that have sufficient sensitivity to reliably identify an active *Bbsl* infection [212,213]. Under these circumstances, clinical manifestations take on increased importance as disease identifiers and clinicians may justifiably arrive at a CLD diagnosis in the absence of direct evidence of an ongoing *Bbsl* infection. In contrast to the ILADS definition, the definition offered by Stricker and Fesler, which is more aptly considered a definition of chronic tick-borne and related diseases, and the Lyme-MSIDS framework may be better suited towards clinical use than research as they encompass the more heterogeneous cohort that is often encountered in clinical practice [27,28]. As such, these two papers could be viewed as complementing the ILADS definition of chronic Lyme disease.

## 9. Conclusions: Summary and Future Directions

Many patients have ongoing manifestations of Lyme disease for prolonged periods of time. ILADS defines chronic Lyme disease (CLD) as a multisystem illness with a wide range of symptoms and/or signs that are either continuously or intermittently present for a minimum of six months. The illness is the result of an active and ongoing infection by any of several pathogenic members of the *Borrelia burgdorferi sensu lato* complex. The infection has variable latency periods and signs and symptoms may wax, wane and migrate. CLD has two subcategories, CLD, untreated (CLD-U) and CLD, previously treated (CLD-PT). The latter requires that CLD manifestations persist or recur following treatment and are present continuously or in a relapsing/remitting pattern for a duration of six months or more. A systematic search of the literature identified cases meeting either the CLD-U or CLD-PT definition that were accompanied by direct evidence of on-going *Bbsl* infection. These cases documented a wide range of manifestations attributable to this active and ongoing infection. This evidence-based definition of CLD is intended to enhance clinician understanding of this infection and to facilitate future research into the diagnostic and therapeutic options of this oftentimes disabling illness. 

## Figures and Tables

**Table 1:** Lists the symptoms, signs and conditions conforming to CLD-U that the investigators attributed to the infection.

CLD-U: Symptoms, Signs, and Conditions in Patients with Direct Evidence of Infection		
**Symptoms and Signs**		
**Constitutional**	**Skin**	**Cardiopulmonary**
Fatigue [71,72,75,106,182,183,184] Fever [75] Weight gain [182]	Atrophic lesions [183] Dry skin [182] Rash, unspecified [185] Skin discoloration [182,183,186,187,188]	Cardiac arrhythmia [138,183,189] Dyspnea [184] Mitral regurgitation [184] Palpitations [184,192] Orthostatic Intolerance [106]
**Head Ears Eyes Nose Throat (HEENT)**	**Musculoskeletal**	**Neuropsychiatric/Neurological**
Blurred vision [76] Double vision [190] Progressive visual loss [72] Decreased visual acuity [191] Nystagmus [190] Photophobia [192,193] Eyelid swelling [192] Facial flushing [75] Facial pain [72,138] Tinnitus [76] Headache [74,75,76,189,194] Stiff neck [74,75] Hearing loss [55,189]	Arthralgia [106,182,186,195,196,197,198] Arthritis [73,75,124,183,198,199,200,201] Joint swelling [72,106,183,186,198,200] Morning stiffness [196] Muscle cramps [197] Muscle weakness [189,202] Myalgia [106,193,202] Muscle atrophy [197,199,202]	Memory difficulties [74,75,106,194] Abnormal taste [76] Dizziness [75,138] Vertigo [76] Decreased sensation [106,197,201] Paresthesias [189,190,196,201] Tingling [197] Pain, generalized [138,197] Pain radicular [76,191] Decreased dexterity [197] Abnormal gait [75,192,197,199] Abnormal balance [138,191] Limb paralysis [183] Spastic paraparesis [197] Positive Babinski [197] Areflexia [191,201] Hyperreflexia [197] Fasciculations [197] Urinary incontinence [197] Decreased concentration [106]
**Conditions**		
Acrodermatitis chronica atrophicans [76,182,185,186,187,188,195,196,202,203] Alzheimer’s disease [204] Anectoderma [205] Carpal tunnel syndrome [189] Cutaneous tumor [206] Dactylitis [207]	Encephalomyelitis [74,75] Encephalopathy [74,75] Endocarditis [184] Epilepsy/seizure [190,194,208] Facial palsy [74,75,193,208] Meningitis [74,75,193] Mitral regurgitation [184] Mycosis fungoides-like rash [185]	Panuveitis [76] Polyarthritis [202] Radiculoneuropathy [74,75] Sensory-motor polyneuropathy [74,197] Sensory neuropathy [75] Synovitis [189,200] Ulcerative keratitis [192]

**Table 2:** Lists the symptoms, signs and conditions conforming to CLD-PT that the investigators attributed to the infection.

CLD-PT: Symptoms, Signs, and Conditions in Patients with Direct Evidence of Infection		
**Symptoms and Signs**		
**Constitutional**	**Skin**	**Cardiopulmonary**
Anorexia [119] Fatigue [22,71,72,113,119,125,136] Fever [113,137,138] Weight loss [22]	Recurrent EM lesions [23,125]	
**HEENT**	**Musculoskeletal**	**Neuropsychiatric/** **Neurological**
Conjunctival irritation [72] Decreased central vision [83] Diplopia [126] Eye pain [72] Photophobia [72] Retro-orbital pain [121] Tinnitus [72] Drooling [22] Fullness in head [125] Headache [71,74,113,126,136,137] Neck pain [22] Stiff neck/torticollis [74]	Arthralgia [23,71,76,83,118,125,126,136,137] Arthritis [73,126] Hand pain [22] Joint swelling [118] Migratory pain [23,126] Muscle stiffness [22] Muscle weakness [139,140] Myalgia [125,126,138,140] Trigger finger [83]	Cognitive dysfunction [119] Poor concentration [125] Memory difficulties [22,74,119,125] Vertigo [121] Dizziness [126] Hypoalgesia [76] Hypoesthesia [76,121] Paresthesias [71,119] Radicular pain [119,137] Cogwheel rigidity [22] Tremors [22]
**Gastrointestinal**	**Genitourinary**	
Vomiting [76]	Nocturia [119] Urge incontinence [22,119] Urgency [119] Urinary frequency [119]	
**Conditions**		
Carpel tunnel syndrome [126] Chorioretinitis [126] Choroiditis [83] Depressed corneal reflexes [121] Encephalitis [126] Encephalomyelitis [74] Encephalomyeloradiculopathy, recurrent [71]	Encephalopathy [74,126] Epilepsy [126] Facial palsy [74] Hepatopathy [126] Hemiparesis [121,126] Meningismus [113] Meningitis [74,126] Mononeuritis multiplex [121] Neuropathy [126] Pericarditis [126]	Pleuritis [126] Radiculitis [126] Radiculoneuropathy [74] Sensory neuropathy [74] Tenosynovitis [83] Trigeminal sensory neuropathy [121] Uveitis [126] Vasculitis [126]

## Abbreviations

*Bb*	*Borrelia burgdorferi*
*Bbsl*	*Borrelia burgdorferi* sensu lato
CDC	Centers for Disease Control
CLD	Chronic Lyme disease
CLD-U	Chronic Lyme disease, untreated
CLD-PT	Chronic Lyme disease, previously treated
CLD-T	chronic Lyme disease, treated
CSF	cerebrospinal fluid
EM	erythema migrans
DNA	deoxyribonucleic acid
HEENT	head, ears, eyes, nose and throat
IgM	immunoglobulin M
IgG	immunoglobulin G
ILADS	International Lyme and Associated Diseases Society
OSP	outer surface protein
PCR	polymerase chain reaction
PLDS	Post Lyme disease Syndrome
PTLDS	Post-Treatment Lyme disease Syndrome
QoL	Quality of Life
TBD	tick-borne disease

## Appendix A

**Table A1:** *Borrelia burgdorferi sensu lato (Bbsl)* Pathogenicity.

**Most Commonly Reported *Bbsl* Pathogens**		
Genospecies	Evidence	Selected References
*B. afzelii*	Culture	Strle (2006) [37]
*B. burgdorferi sensu stricto*	Culture	Steere (1984) [34] Nowakowski (2009) [35] Smith (2002) [36]
*B. garinii*	Culture	Strle (2006) [37]
**Less Commonly Reported *Bbsl* Pathogens**		
*B. americana*	PCR	Clark (2013) [50]
*B. andersonii*	PCR	Clark (2013) [50]
*B. bavariensis*	PCR	Markowicz (2015) [48] Tijsse-Klasen (2013) [49]
*B. bissettii*	PCR	Golovchenko (2016) [45] Rudenko (2008) [46] Rudenko (2009) [47]
*B. lusitaniae*	PCR	Collares-Pereira (2004) [44]
*B. mayonii*	PCR	Pritt (2016) [43]
*B. spielmanii*	Culture & PCR	Maraspin (2006) [38]
	PCR	Fingerle (2008) [39] Földvári (2005) [40]
*B. valaisiana*	Immunoblot	Ryffel (1999) [41]
*B. sp A14S*	PCR	Wang (1999) [42]
***Bbsl *** **Genospecies Without Established Pathogenicity**		
*B. californiensis*		Postic (2007) [54]
*B. carolinensis*		Foley (2014) [55]
*B. japonica*		Rudenko (2011) [51]
*B. kurtenbachii*		Margos (2010) [56]
*B. lanei*		Margos (2017) [57]
*B. sinica*		Rudenko (2011) [51]
*B. tanuki*		Rudenko (2011) [51]
*B. turdi*		Rudenko (2011) [51]
*B. yangtze*		Rudenko (2011) [51]
